# The Effects of Adverse Events and Associated Costs on Value-Based Care for Metastatic Pancreatic Ductal Adenocarcinoma

**DOI:** 10.36469/001c.124367

**Published:** 2024-12-18

**Authors:** Prachi Bhatt, Jared Hirsch, Paul Cockrum, George Kim, Gabriela Dieguez

**Affiliations:** 1 Milliman (United States); 2 Ipsen (United States); 3 George Washington University

**Keywords:** metastatic pancreatic ductal adenocarcinoma, Medicare, pancreatic cancer, adverse events, total cost of care, value-based care

## Abstract

**Background:** Rising oncology healthcare costs have led to value-based care reimbursement models that coordinate care and improve quality while reducing overall spending. These models are increasingly important for traditional Medicare and other payers. **Objectives:** To compare the incidence of adverse events (AEs), AE-associated excess costs, and total cost of care (TCOC) of 3 cohorts receiving first-line treatment for metastatic pancreatic ductal adenocarcinoma (mPDAC). **Methods:** We conducted a retrospective analysis of administrative claims data from 2018 to 2022 using the Medicare 100% Research Identifiable Files. We examined 3 cohorts receiving mPDAC treatment: FOLFIRINOX (FFX) (oxaliplatin, irinotecan, leucovorin, 5-FU bolus and infusion); modified FFX, (5-FU infusion only); and gemcitabine/nab-paclitaxel (gem/abrax). We compared the incidence of clinically significant AEs, TCOC, components of TCOC, and costs related to AEs/treatment toxicity. **Results:** Patient AE rates ranged from 6.2% to 51.7%. AEs occurred more frequently in patients receiving FFX with all 4 components. Patients receiving brand name gem/abrax had lower rates of febrile neutropenia (6.2%) and neutropenia (22.2%) than those receiving FFX with no 5-FU bolus (febrile neutropenia, 9.9%; neutropenia, 36.9%) and FFX with all 4 components (febrile neutropenia, 6.9%; neutropenia, 30.4%). Rates of most nonhematologic AEs were higher in patients receiving FFX with all 4 components, with diarrhea occurring in 28.3%, abdominal pain in 31.5%, and nausea/vomiting in 41.5% of patients. TCOC was lower in the gem/abrax cohort: 6505vsFFXwithno5−FUbolus(6995) and FFX with all 4 components (7142)peradministration.ThedevelopmentofanystudiedhematologicAEwasassociatedwithameanexcesscostof5993 per administration, while the development of any studied nonhematological AE was associated with a mean per-administration excess cost of $3665. **Discussion:** Treatment decisions intended to minimize chemotherapy costs may lead to suboptimal decisions if the goal is to reduce TCOC. Our research suggests FFX is more costly than gem/abrax (TCOC per administration). Patients receiving gem/abrax were older and had higher baseline Charlson Comorbidity Index scores; however, other factors may be important in driving cost differences. **Conclusions:** Irrespective of drug cost, chemotherapy leading to a significant increase in AEs is associated with higher TCOC.

## INTRODUCTION

Pancreatic cancer is among the deadliest diseases and currently is the third leading cause of cancer deaths in the United States.^1^ Pancreatic ductal adenocarcinoma (PDAC), the most common type of pancreatic cancer, represents approximately 90% of all pancreatic cancers.^1^ An estimated 62 200 new cases of PDAC are diagnosed each year in the United States, and the disease results in approximately 48000 deaths each year.^1^ A particularly aggressive cancer, PDAC is estimated to have a 5-year overall survival rate of 11%.^1^ US and global data suggest the incidence and prevalence of PDAC are steadily rising, in part because of the increasing incidence of obesity, diabetes, and other conditions that play a role in the development of pancreatic cancer.^1^

In contrast to many cancers, PDAC lacks methods of early detection; consequently, most affected individuals (>50%) are diagnosed at an advanced stage of disease.^2^ Surgical resection is the only potentially curative intervention for PDAC; however, only 15% to 20% of all patients with PDAC are diagnosed with surgically resectable disease.^3^ Although for many years patients who were ineligible for resection were treated with monotherapy, more recently, combination treatments have produced more successful outcomes.^4,5^ According to the 2021 National Comprehensive Cancer Network (NCCN) guidelines, first-line (1L) treatment recommendations for patients with metastatic PDAC (mPDAC) who have good performance status include the use of FOLFIRINOX, modified FOLFIRINOX, or gemcitabine + albumin bound paclitaxel. For patients with poor performance status, recommendations include gemcitabine monotherapy, capecitabine monotherapy, or continuous infusion 5-fluorouracil (5-FU).^6^

Research has suggested while combination therapies may improve outcomes for patients with PDAC, such therapies also increase the risk of experiencing adverse events (AEs).^7^ Real-world data suggests a higher rate of therapy discontinuation occurs due to AEs compared with discontinuation rates observed in landmark clinical trials.^8,9^ Moreover, AEs are often associated with substantial costs and less favorable outcomes.^10–13^ While generic regimens may seem preferable when evaluated from a chemotherapy cost perspective, regimens associated with higher rates of AEs may increase the total burden and cost of care.^14^ As prevalence of mPDAC increases and new therapies become available, there is a need to quantify the benefit of new therapies from a value-based approach.

The use of value-based care reimbursement models has grown in the United States with the introduction of the Affordable Care Act as an alternative to the prevailing fee-for-service provider reimbursement system. Value-based care reimbursement frameworks give providers incentives to manage both the total cost and outcomes of patient care. The Centers for Medicare and Medicaid Services (CMS) has introduced several value-based care reimbursement programs affecting Medicare providers, which have been increasingly adopted by payers and providers in the commercial and managed Medicare markets.

Accountable care organizations (ACOs) are one of the most broadly implemented value-based care reimbursement models in which providers assume financial risk for the cost of care and the outcomes of their patients.^15^ ACOs have grown considerably both in number as well as in the number of covered lives.^16^ As of the beginning of 2022, there were 1010 ACOs covering a total of 32 million lives.^17^ There are 2 types of risk when discussing financial risk arrangements in value-based care: upside risk, in which there is uncertainty in the potential gains, and downside risk, in which there is uncertainty in the potential losses.^18^ In an analysis of ACO contract types, 33% of ACOs had at least 1 contract that included downside risk.^15^ In such arrangements, providers that deliver quality care at a lower cost may be eligible to receive a payment from CMS, while participants who increase overall spending may owe a payment to CMS. This type of arrangement provides strong incentives for delivering value-based care, with an emphasis on responsibility for managing the total cost of all care, rather than a focus on a single aspect of care. Another program, the Oncology Care Model (OCM), was introduced in 2015 with the aim of creating a coordinated approach to oncology treatment intended to improve outcomes while reducing unneeded costs and care.^19^ This model has been adopted by commercial payers as well.^20,21^ A successor program to the OCM, the Enhancing Oncology Model (EOM), is focused on optimizing patient care while avoiding unnecessary costs. Participating providers in EOM assume financial and performance accountability for the treatment of patients with common cancer types receiving systemic chemotherapy. Providers receive an episode-based payment that financially incentivizes high-quality, coordinated care.^19^

In several types of cancer, the cost of care has been found to increase substantially when patients experience AEs.^10–12^ Examples of AEs in the OCM and EOM model are anemia, dehydration, diarrhea, emesis, fever, nausea, neutropenia, pain, pneumonia, or sepsis.^22^ AEs can negatively affect both clinical outcomes and quality of life, and disrupt treatment plans, often leading to therapy changes (such as dose delays or reductions), lower adherence, and even discontinuation.^8,23^ A growing number of studies are indicating the costs associated with the management of AEs or complications should be considered in the costs of an intervention.^10^ Chemotherapy-associated AEs place a significant burden on patients, with a direct effect on symptoms and health-related quality of life and function; they also compromise treatment intensity and continuation, and potentially increase risk of cancer recurrence. Knowledge about the long-term effects of certain early AEs remains limited. For example, cancer-related fatigue, febrile neutropenia (often with serious hospitalizations owing to infection), and chemotherapy-induced peripheral neuropathy most commonly manifest early during treatment and yet can have persistent long-term effects for survivors years after the initial cancer treatment or can be ongoing for those cancer survivors who remain on treatment.^24^

### Objectives

The incidence and financial burden of AEs have not been evaluated within the context of mPDAC. This study aims to quantify the incidence of AEs and total cost of care (TCOC) for real-world patients receiving 1L therapies for mPDAC. We compare treatment with 1L FOLFIRINOX (FFX), 1L modified FFX, and 1L gemcitabine/abraxane (gem/abrax) to determine whether there is a significant difference in incidence rates of clinically meaningful AEs and the TCOC (including costs associated with chemotherapies and AEs), as a proxy for the more comprehensive decision-making associated with value-based care.

## METHODS

We conducted a retrospective analysis of administrative claims data for patients with mPDAC using the Medicare 100% Research Identifiable Files (RIF) database for years 2018-2022. The RIF claims files contain all Medicare paid Fee-For-Service claims generated for all Medicare beneficiaries in the United States for all Part A, B, and D services. Information in the 100% RIF claims files includes diagnosis codes, procedure codes, DRG codes, site of service information, and beneficiary information, including age, geographic census region, and dual eligibility status.

Index date was defined as the first observed day of chemotherapy following the initial diagnosis of mPDAC. Treatment cohorts were assigned based on drugs present within 2 days of index: (1) FFX with all 4 component drugs (oxaliplatin, irinotecan, leucovorin, 5-FU bolus and infusion); (2) FFX with no 5-FU bolus; and (3) gem/abrax. These agents represent NCCN guideline–recommended 1L therapy for mPDAC. Any other modifications of FFX, such as omitting 5-FU altogether, omitting leucovorin, administration of the 5-FU bolus only, were excluded from this analysis as sensitivity testing determined these patients in total comprised less than 4% of the overall total incident mPDAC population and do not reflect NCCN recommended treatment regimens.

Patients in the study had at least 2 diagnosis codes for malignant neoplasm of pancreatic duct (ICD-10 C25.3), recorded on different dates, *and* at least 1 diagnosis of metastasis (ICD-10 codes C77.xx, C78.xx, C79.xx, C80.0, and/or C80.1) anytime on or after the first pancreatic cancer diagnosis to be eligible for inclusion in the study. Patients under the age of 18, with evidence of end-stage renal disease, or with missing age or gender were excluded from the study. Only patients receiving 1L therapy were included in this study. Patients were followed until death, disenrollment, therapy abandonment (defined as evidence of no therapy for 42 days) or evidence of second-line chemotherapy administration, whichever came first. If a patient transitioned from an initial treatment cohort to another cohort, only data from the initial cohort was included in this analysis and these patients were not analyzed for chemotherapy resistance or progression. Performance status is not available in retrospective claims data and thus was not evaluated in this analysis.

The primary outcomes were incidence of AEs, TCOC, and excess costs associated with the various AEs. Additional outcomes were aimed at evaluating efficacy and outcomes metrics and included time to next administration of the same chemotherapy regimen (time to same treatment, which was used to determine therapy continuation), percentage of patients who die while receiving 1L treatment, and rates of healthcare resource utilization including inpatient admissions and readmissions, outpatient visits, emergency department (ED) visits, and hospice encounters.

Costs were defined as allowed costs and were calculated by service category for each administration. An administration begins at the first chemotherapy administration and lasts until the next observation of chemotherapy administration. If a second administration was not observed, costs were calculated until disenrollment, death, therapy abandonment, or evidence of second-line therapy.

Incidence of both hematologic adverse events (hAEs) and nonhematologic adverse events (nhAEs) were determined by the presence of ICD-10 codes for each AE for the duration of 1L treatment. Hemotologic AEs included anemia, thrombocytopenia, neutropenia, and febrile neutropenia and nhAEs included diarrhea, nausea/vomiting, fatigue, neuropathy, and abdominal pain. These AEs were selected as they represent the commonly seen AEs associated with 1L mPDAC treatment.^16^ We compared outcomes across the 3 cohorts to assess these 1L therapies from the perspective of value-based care reimbursement models.

Generic Abraxane became available in April 2022. Since the introduction of generic therapies during our study period could have had a substantive impact on chemotherapy drug costs, we summarized uptake and cost of brand vs generic Abraxane to understand the impact of the introduction of generic Abraxane on the TCOC.

## RESULTS

**Figure 1.**
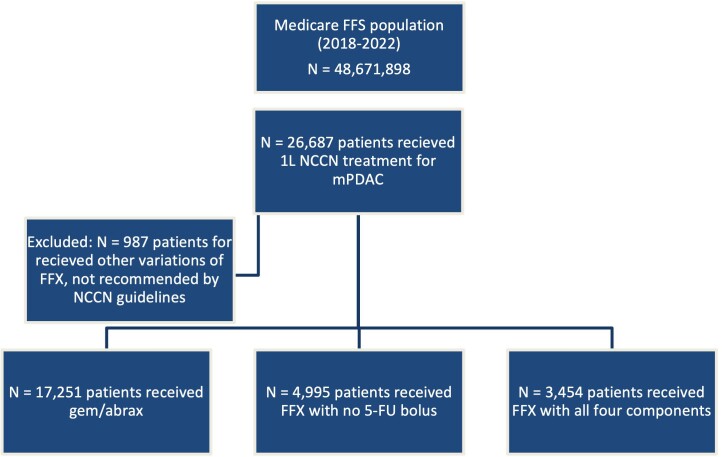
Study Population Abbreviations: FFS, Fee-For-Service; FFX, 1LFOLFIRINOX; mPDAC, metastatic pancreatic ductal adenocarcinoma; NCCN, National Comprehensive Cancer Network.

There were a total of 25 700 patients included in this study, 13% (n = 3454) receiving FFX with all 4 components, 19% (n = 4995) receiving FFX with no 5-FU bolus, and 67% (n = 17,251) receiving gem/abrax (**Figure 1**).

Average age at index was 69.9 years for FFX with all 4 components, 70.4 years for FFX with no 5-FU bolus, and 73.1 years for the gem/abrax cohort (**Table 1**). Average Charlson Comorbidity Index (CCI) score at index was 3.8 for FFX with all 4 components, 4.0 for FFX with no 5-FU bolus, and 4.2 for the gem/abrax cohort. The average number of administrations per patient was 5.9 for FFX with all 4 components, 5.8 for FFX with no 5-FU bolus, and 8.6 for the gem/abrax cohort. The average duration of 1L treatment was 130 days for FFX with all 4 components, 124.9 days for FFX with no 5-FU bolus, and 138.5 days for the gem/abrax cohort. Patients in the gem/abrax cohort had the lowest survival, with 86.3% surviving while receiving gem/abrax compared with 91.3% for patients receiving FFX with all 4 components and 93.3% survival for patients receiving FFX with no 5-FU bolus.

**Table 1.** Patient Demographics

	**Cohort**				
	**Subtotal Study Population**	**FFX – All 4 Components**	**FFX – No Bolus**	**Gem Abraxane**	**All Other FFX^a^**
No. of patients	25,700	3454	4995	17,251	987
Distribution of patients, %	100	13	19	67	4
Demographic characteristics					
Female, %	48	43	46	50	47
*P* value		<.001	<.001	*–*	*–*
Dual-eligible, %	14	14	12	14	13
*P* value		<.001	<.001	–	–
Average age at index date (years)	72.1	69.9	70.4	73.1	70.1
*P* value		<.001	<.001	*–*	*–*
Median age at index date (SD)	70 (6.2)	70 (5.8)	73 (6.9)	70 (5.7)	72 (6.7)
Average CCI score at index date	4.1	3.8	4.0	4.2	3.7
*P* value		<.001	<.001	*–*	*–*
Median (SD) CCI score at index date	3 (3.1)	3 (3.0)	3 (3.3)	3 (3.0)	3 (3.2)
Treatment characteristics					
Total No. of administrations	198,097	20,502	29,134	148,461	5149
Average No. of administrations per patient	7.7	5.9	5.8	8.6	5.2
Average time to next administration,^b^ days	17.5	21.9	21.4	16.1	22.2
Patients discontinuing treatment, %	1.0	0.7	0.4	1.2	0.0
Average length of treatment, days	134.7	130.0	124.9	138.5	115.9
Patients who survived while receiving 1L treatment, %	88.3	91.3	93.3	86.3	92.4

Reference population: gem Abraxane.

^a^Not included in the study population.

^b^Average time to next administration: Mean days elapsed between administrations of the same treatment.

Abbreviations: 1L, first-line; CCI Charlson Comorbidity Index; FFX, FOLFIRINOX (oxaliplatin, irinotecan, leucovorin, 5-FU bolus and infusion).

On average, patients in the study incurred 0.26 inpatient admissions per patient year. Average admissions per patient year were 0.22 for FFX with all 4 components, 0.19 for FFX with no 5-FU bolus, and 0.29 for the gem/abrax cohort (**Table 2**). Average times to hAEs and nhAEs from first administration were 74 days and 58 days for FFX with all 4 components, 72 days and 57 days for FFX with no 5-FU bolus, and 58 days and 51 days for the gem/abrax cohort, respectively (**Table 2**).

**Table 2.** Toxicity and Adverse Events

			**Cohort**		
	**Subtotal Study Population**	**FFX – All 4** **Components**	**FFX – No** **Bolus**	**Gem Abraxane**	**All Other FFX^a^**
HCRU per patient-year					
Average No. of admissions	0.26	0.22	0.19	0.29	0.26
Average No. of readmissions	0.08	0.06	0.05	0.09	0.08
Average No. of IP/OP within 30 days	0.15	0.15	0.13	0.16	0.15
Average No. of ED/obs visits	1.30	0.65	0.50	1.69	1.30
Time to AE, days from first administration					
Average time to IP	66	62	58	69	61
Average time to ER/obs	66	67	55	68	52
Average time to heme AE	63	74	72	58	80
Average time to non-heme AE	53	58	57	51	63

Abbreviations: AE, adverse event; ED, emergency department; heme, hematologic; IP, inpatient admission; obs, observation; non-heme, nonhematologic; OP, outpatient visit.

^a^Not included in the study population.

**Figure 2.**
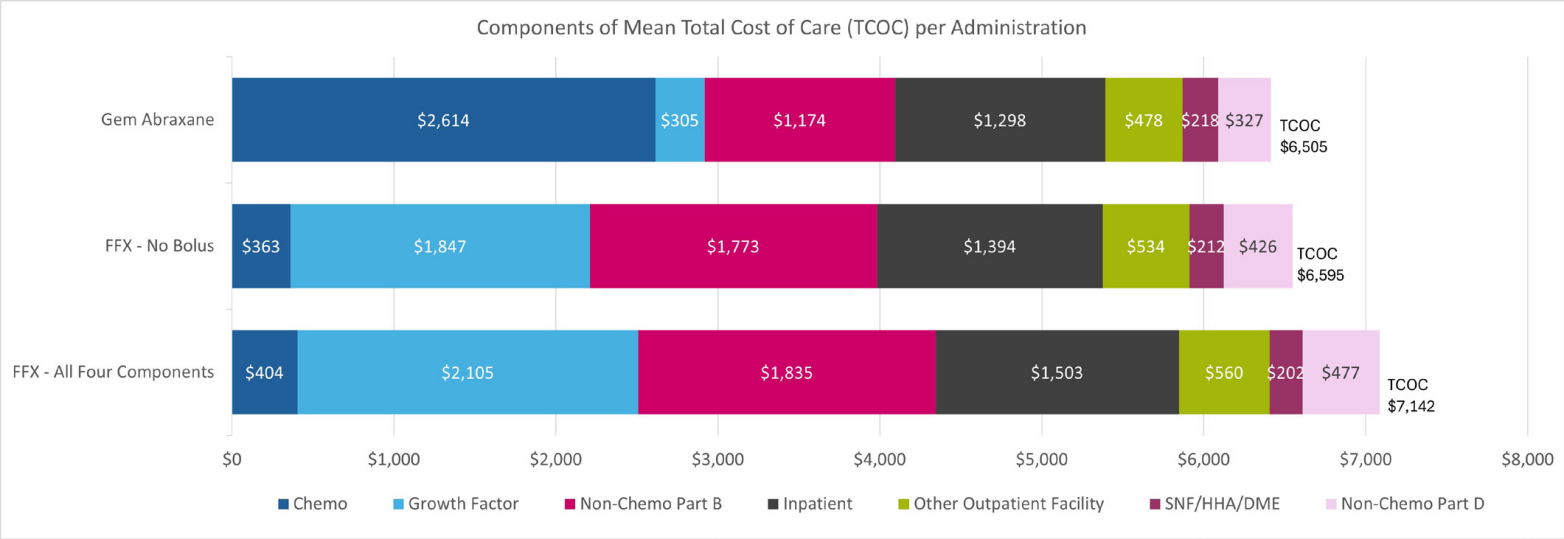
Components of Total Cost of Care per Administration Abbreviations: DME, durable medical equipment; HHA, home health aide; SNF, skilled nursing facility.

The cohorts we examined were remarkably similar with regard to TCOC per-administration, despite substantial differences in chemotherapy costs. The mean TCOC per administration was $7142 for FFX with all 4 components, $6595 for FFX with no 5-FU bolus, and $6505 for the gem/abrax cohort (**Figure 2**). The largest cost driver for TCOC for FFX with no 5-FU bolus and FFX with all 4 components was growth factor costs at $1847 and $2105 respectively, whereas for gem/abrax it was chemotherapy costs, which accounted for $2614. The time to next administration was, on average, 21.9 days for FFX with all 4 components, 21.4 days for FFX with no 5-FU bolus, and 16.1 days for the gem/abrax cohort. Patients treated with gem/abrax received more administrations per month (mean, 1.9) than patients treated with FFX with no 5-FU bolus or FFX with all 4 components (mean, 1.4). Of note, we did not attempt to identify patients in 3 times vs twice-monthly regimens among the gem/abrax cohort, as this information is not reliable in administrative claims data. The TCOC per patient per month for patients receiving gem/abrax 3 times monthly would be higher than both those receiving gem/abrax twice monthly and those receiving FFX regimens (either with all 4 components or without 5-FU bolus), which consists of 2 administrations per month. The TCOC on a per patient per month basis are provided in the **Online Supplementary Material**.

We observed an increase in generic Abraxane prescribing starting in April 2022 (when it first became available), with a peak generic utilization rate of 31% in October 2022. Due to a national shortage, generic utilization rates subsequently dropped to 23% in November 2022. Our analysis of RIF files through December 2023 suggests these rates have not since recovered and have remained consistently below 5% since June 2023. Costs of both branded and generic products have remained consistent with each other. Cost per unit was approximately $14 for both branded and generic products from January 2022 until September 2022, when they both decreased to approximately $12 per unit, where they have remained. Because of the low uptake rates, our analysis reflects minimum impact of generic Abraxane uptake.

**Figure 3.**
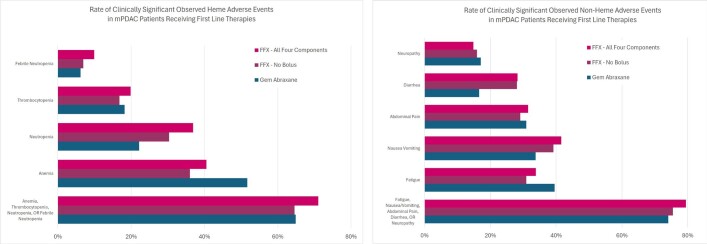
Rates of Clinically Significant Observed Adverse Events Abbreviations: AE, adverse event; FFX, FOLFIRINOX (oxaliplatin, irinotecan, leucovorin, 5-FU bolus and infusion).

Clinically significant observed AE rates are reported in **Figure 3** for each patient cohort. Patients receiving gem/abrax had lower rates of febrile neutropenia occurring in 6.2% of patients, and neutropenia occurring in 22.2%, compared with those receiving FFX with no 5-FU bolus (febrile neutropenia 9.9%, neutropenia 36.9%) and FFX with all 4 components (febrile neutropenia 6.9%, neutropenia 30.4%). Rates of most clinically significant observed nonhematological adverse events were overall higher in patients receiving FFX with all 4 components with diarrhea occurring in 28.3%, abdominal pain in 31.5%, and nausea/vomiting in 41.5%.

**Figure 4.**
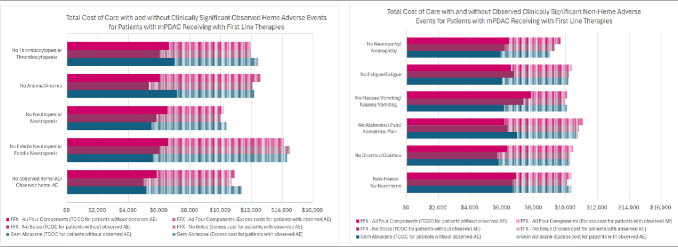
Excess Costs of Observed Adverse Events Abbreviations: AE, adverse event; heme, hematologic; non-heme, nonhematologic.

We also evaluated the per-administration excess costs for patients that develop an AE of interest (**Figure 4**). We determined patients who develop anemia, thrombocytopenia, neutropenia, or febrile neutropenia incur an excess mean per-administration cost of $5993 compared with patients who had no evidence of those AEs. Patients who developed diarrhea, abdominal pain, nausea/vomiting, fatigue, or neuropathy incurred an excess mean per-administration cost of $3665 compared with patients who did not develop any of those AEs.

Observation of an AE was based on ICD-10 codes present in retrospective claims data for the listed AEs only.

## DISCUSSION

Our study suggests the cost of chemotherapies for the treatment of mPDAC are not the main driver of costs for patients receiving 1L therapy. In fact, other components of care, in particular growth factor administrations, nonchemotherapy Part B costs, and inpatient services contribute more to the TCOC than chemotherapy drug costs. The high cost associated with inpatient and outpatient facilities observed point to the significant burden associated with the development of AEs. We determined that patients receiving FFX experienced AEs at a higher rate than patients receiving gem/abrax. Although gem/abrax has higher chemotherapy costs, the TCOC per-administration was higher in patients receiving FFX, suggesting that AEs play a large role in driving costs. This finding highlights the importance of evaluating all components of TCOC in patients receiving treatment associated with significant AEs. This finding also points to an opportunity for value-based care reimbursement frameworks focused on improving patient outcomes and lowering TCOC.

Patients receiving gem/abrax had lower survival rates and higher rates of hospital stays and ED visits. These results may be explained by the higher age and CCI observed at baseline in the gem/abrax cohort compared with the 2 FFX cohorts. The lower rate of AEs and lower TCOC observed in patients receiving gem/abrax, despite those patients being older and more comorbid at baseline, suggests the impact of drug toxicity on TCOC.

Value-based care reimbursement models are an increasingly important focus not only for traditional Medicare, but also for other payers, including commercial and Medicare Advantage payers as well as managed Medicaid. The cost of chemotherapy alone is not a good benchmark for comparing therapies under value-based care reimbursement models. Instead, TCOC and patient outcomes, such as incidence of AEs, overall survival, rates of inpatient admission/readmission, and ED visits, are better benchmarks for assessing the true cost of therapies. Implicit in an evaluation of TCOC is the balance of patient out-of-pocket costs and access to care, which are areas where future research may be warranted.

## Limitations

We rely on the use of administrative claims submitted to CMS for the purposes of reimbursement. This analysis may not be generalizable outside of a Medicare population. Retrospective administrative data are not clinical in intent and does not contain information on stage, performance status, progression-free survival (which require information on clinical or radiologic progression which is unavailable in claims data) or other clinically significant variables. Patients in this study were observed in a retrospective manner and were not randomized into treatment groups; inherent differences in patient characteristics and treatment biases may be present and have not been accounted for. We relied on the observation of claims for chemotherapy within a 48-hour period to determine treatment cohorts, any inaccuracies in the date of the claim submitted may impact patient characterization. Additionally, incidence of AEs was based on the occurrence of an AE that led to patient contact with the healthcare system and a subsequent claim coded with the event. We determined the AE incidence rates for only the listed clinically relevant AEs; other AEs could have occurred, but we do not report these rates. While grade of AEs is not available in retrospective claims data, the resultant claim with the event signifies the patient experienced, at minimum, an event that was serious enough to seek care. Any over-the-counter care or care in accordance with a previously determined treatment plan will not be captured by this analysis.

## CONCLUSION

Our findings suggest patients with mPDAC treated with 1L therapies experience AEs at high rates, ranging from 6.2% up to 51.7%. While there were differences in the components of TCOC (chemotherapy, growth factor, nonchemotherapy Part B, inpatient, outpatient facility, SNF/HHA/DME, and Part D services), including higher chemotherapy drug costs for gem/abrax, TCOC was lower in the gem/abrax cohort at $6505 per administration compared with FFX with no 5-FU bolus which was $6995 per administration, and FFX with all 4 components was $7142 per administration. This difference was present across the entire treatment cohort, even those with low-income status.

AEs occurred more frequently in patients receiving FFX with all 4 components. Compared with chemotherapy costs, mean excess per-administration costs associated with AEs, which were $5993 if a hematologic AE developed and $3665 if a nonhematologic AE developed, are consequential. Our research demonstrates FFX, which is a generic regimen, is more costly per-administration than gem/abrax, which, in our study, had generic utilization rates of only 23% to 31%. The higher observed TCOC was despite lower chemotherapy costs for the FFX cohort along with a mean age 3 years younger and a lower baseline CCI (*P* < .001 for both measures). Despite differences in chemotherapy costs, regimens that lead to a significant increase in AEs/toxicity lead to a higher excess cost due to the increased cost burden of AEs.

### Disclosures

Gabriela Dieguez, Prachi Bhatt, and Jared Hirsch received consulting fees from Ipsen Biopharmaceuticals to perform this study.

## Supplementary Material

Online Supplementary Material
