# Supplementary material for: The Effects of Adverse Events and Associated Costs on Value-Based Care for Metastatic Pancreatic Ductal Adenocarcinoma
Source: J Health Econ Outcomes Res. 2024 Dec 18;11(2):161–7. doi: 10.36469/001c.124367 (PMC11664866; doi:10.36469/001c.124367)
Supplement: Online Supplementary Material [file jheor_2024_11_2_124367_256236.pdf]

Journal of Health Economics and Outcomes Research

Online Supplementary Material

Title Case. *JHEOR*. 2024;11(2):161-167. doi:10.36469/jheor.2024.124367

Figure S1: Components of Total Cost of Care per Patient per Month

This supplementary material has been provided by the authors to give readers additional information about their work.

This is an open-access article distributed under the terms of the Creative Commons Attribution 4.0 International License (CCBY-4.0). View this license's legal deed at http://creativecommons.org/licenses/by/4.0 and legal code at http://creativecommons.org/licenses/by/4.0/legalcode for more information.

Figure S1. Components of Total Cost of Care per Patient per Month

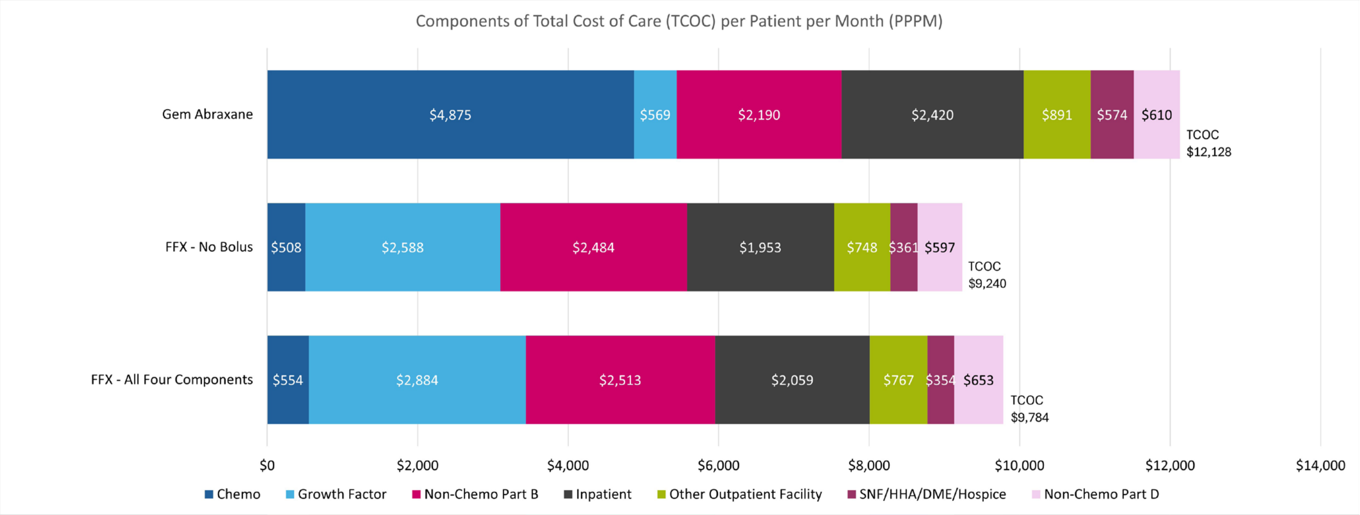

Abbreviations: DME, durable medical equipment; HHA, home health aide; SNF, skilled nursing facility.
